# An ENU-induced splice site mutation of mouse Col1a1 causing recessive osteogenesis imperfecta and revealing a novel splicing rescue

**DOI:** 10.1038/s41598-017-10343-9

**Published:** 2017-09-15

**Authors:** Koichi Tabeta, Xin Du, Kei Arimatsu, Mai Yokoji, Naoki Takahashi, Norio Amizuka, Tomoka Hasegawa, Karine Crozat, Tomoki Maekawa, Sayuri Miyauchi, Yumi Matsuda, Takako Ida, Masaru Kaku, Kasper Hoebe, Kinji Ohno, Hiromasa Yoshie, Kazuhisa Yamazaki, Eva Marie Y. Moresco, Bruce Beutler

**Affiliations:** 1 Division of Periodontology, Department of Oral Biological Science, Niigata University Graduate School of Medical and Dental Science, Niigata, Japan; 2 Division of Medical Genetics, Department of Medicine, University of California San Diego, La Jolla, California, United States of America; 3 Research Center for Advanced Oral Science, Niigata University Graduate School of Medical and Dental Sciences, Niigata, Japan; 4 Department of Developmental Biology of Hard Tissue, Graduate School of Dental Medicine, Hokkaido University, Sapporo, Japan; 5 Centre d’Immunologie de Marseille-Luminy, Aix Marseille Université, INSERM, CNRS, 13288 Marseille, France; 6 Division of Bio-Prosthodontics, Niigata University Graduate School of Medical and Dental Sciences, Niigata, Japan; 7 Division of Immunobiology, Cincinnati Children’s Hospital Research Foundation, Cincinnati, Ohio, United States of America; 8 Division of Neurogenetics, Center for Neurological Diseases and Cancer Nagoya University Graduate School of Medicine, Nagoya, Japan; 9 Laboratory of Periodontology and Immunology, Department of Oral Health and Welfare, Faculty of Dentistry, Niigata University, Niigata, Japan; 10 Center for the Genetics of Host Defense, University of Texas Southwestern Medical Center, Dallas, Texas United States of America

## Abstract

GU-AG consensus sequences are used for intron recognition in the majority of cases of pre-mRNA splicing in eukaryotes. Mutations at splice junctions often cause exon skipping, short deletions, or insertions in the mature mRNA, underlying one common molecular mechanism of genetic diseases. Using N-ethyl-N-nitrosourea, a novel recessive mutation named *seal* was produced, associated with fragile bones and susceptibility to fractures (spine and limbs). A single nucleotide transversion (T → A) at the second position of intron 36 of the *Col1a1* gene, encoding the type I collagen, α1 chain, was responsible for the phenotype. *Col1a1* ^*seal*^ mRNA expression occurred at greatly reduced levels compared to the wild-type transcript, resulting in reduced and aberrant collagen fibers in tibiae of *seal* homozygous mice. Unexpectedly, splicing of *Col1a1* ^*seal*^ mRNA followed the normal pattern despite the presence of the donor splice site mutation, likely due to the action of a putative intronic splicing enhancer present in intron 25, which appeared to function redundantly with the splice donor site of intron 36. *Seal* mice represent a model of human osteogenesis imperfecta, and reveal a previously unknown mechanism for splicing “rescue.”

## Introduction

Type I collagen is a major structural component of mammalian bone, constituting >90% of bone organic components. Biosynthesis of type I collagen is a long and complex process, including intra- and extracellular posttranslational modifications. In brief, two type I collagen α1 chains and one α2 chain supercoil into a triple helix structure and are enzymatically cleaved to form mature type I collagen. Intra- and inter-molecular covalent cross-links between α chains of mature type I collagen mediate formation of collagen fibrils to organize bone^1^.

The bulk of each α chain consists of a repeating three-amino acid unit, Gly-X-Y, that is necessary for triple helix formation. Mutations in the pro-α1 and -α2 chains, encoded by *COL1A1* and *COL1A2*, can cause defects in type I collagen synthesis or assembly resulting in osteogenesis imperfecta (OI), a genetic disorder characterized by bone fragility and deformity, blue sclera, short stature, dentinogenesis imperfecta, and hearing loss. To date, more than 1,500 mutations in *COL1A1* and *COL1A2* have been identified in patients with OI, among which nonsense, frame shift, and splicing mutations often cause quantitative deficiency in the pro-α chains, whereas missense mutations lead to aberrant pro-α chains that exert a dominant negative effect on collagen synthesis^2–4^. The most common missense mutations are glycine substitutions within the Gly-X-Y in the triple helix. More severe clinical phenotypes manifest in OI patients with helical glycine mutations than those with other mutations causing quantitative procollagen deficiencies^5,6^.

*COL1A1* and *COL1A2* contain approximately 52 intronic sequences that need to be precisely excised to generate mature mRNA, making these genes particularly susceptible to splicing mutations^7,8^. Accurate splicing depends on splice sites, conserved sequence elements positioned at the 5′ ends (5′ss or splice donor sites) and 3′ ends of introns (3′ss or splice acceptor sites) that are recognized by components of the spliceosome^9^. Greater than 99% of mammalian introns, including all of those in *COL1A1*, are spliced by the major (U2-dependent) spliceosome and have as their terminal dinucleotides GU (5′ end) and AG (3′ end)^10^. These dinucleotides are invariant in splice sites of major (U2-type) introns and are therefore considered critical elements of them. In contrast, flanking nucleotides (up to 3 bp into the adjacent exon and 8 bp into the intron for 5′ss) may deviate from consensus sequences resulting in variation in splice site strength^11^. Splice donor and acceptor site mutations can lead to exon skipping, use of cryptic splice sites, and/or insertions/deletions; many human diseases stem from such defects caused by splice site mutations, and their mechanisms have been well documented^12^. Importantly, the effects of splice site mutations are determined in part by the order and rate of intron removal from the pre-mRNA^13,14^. Exon skipping mutations causing lethal or moderate phenotypes of OI have been identified in both splice donor and acceptor consensus sequences in *COL1A1* and *COL1A2* genes^15,16^. Novel causative mutations for OI continue to be identified^17^.

Here we report the identification and characterization of a novel N-ethyl-N-nitrosourea (ENU)-induced *Col1a1* mutation named *seal* that causes OI in homozygous mice. Analysis of the effects of the donor splice site mutation led to the discovery of a regulatory element for splicing within intron 25, acting on the excision of intron 36.

## Results

### Identification of the seal phenotype and its genetic cause

**Figure 1:**
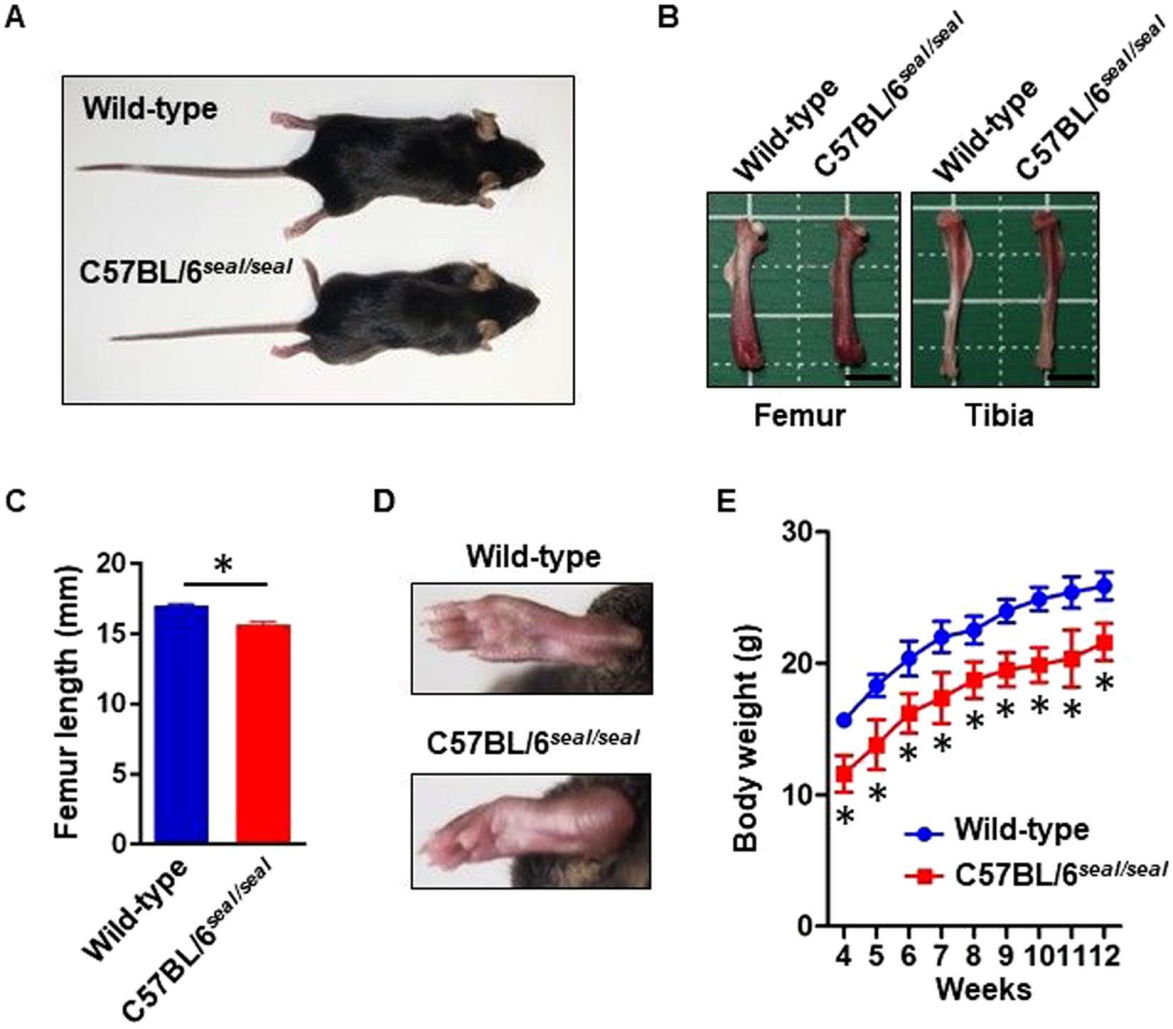
Phenotype of *seal* mutant mice. All the specimens were from male mice. (**A**) Wild-type and *seal* homozygous mice (12 weeks of age). (**B**) Femur and tibia of a 12-week-old *seal* homozygous mouse. They are abnormal in shape and shorter than the corresponding wild-type bones. Bone marrow is visible through thin cortical bone of *seal* homozygous animals. Scale: length of smallest grid square, 5 mm. (**C**) Femur length. N = 5 mice per genotype. (**D**) Swollen and deformed foot of a *seal* homozygote. (**E**) Body weight of age-matched mice. N = 5 mice per genotype. In C and E, results are expressed as mean ± SD.

The recessive *seal* phenotype was initially recognized as a defect of hind limb movement induced by grasping the loose skin over the nape of the neck, as is commonly practiced during routine handling of laboratory mice. Once triggered in this manner, the hind legs become paralyzed for a period of about 8 days before they regain function. However, most of the mice still display a residual “seal-like” gait after the recovery. Homozygous *seal* mice show shortened limbs due to a reduction in the length of the long bones relative to that of wild-type littermates (Fig. 1A–C). About 50% of *seal* homozygotes also have swollen heels and foot pads, occasionally with deformed feet due to pathologic fracture (Fig. 1D). Necropsy of *seal* mice that showed abnormal locomotion revealed spinal bone fracture that presumably caused hind limb paralysis. Body weight of *seal* mice was reduced 8% compared to those of wild-type mice throughout the period of rapid growth between 6 and 12 weeks of age (Fig. 1E). All described phenotypes were transmitted in a recessive manner and heterozygotes were indistinguishable from wild-type mice.

**Figure 2:**
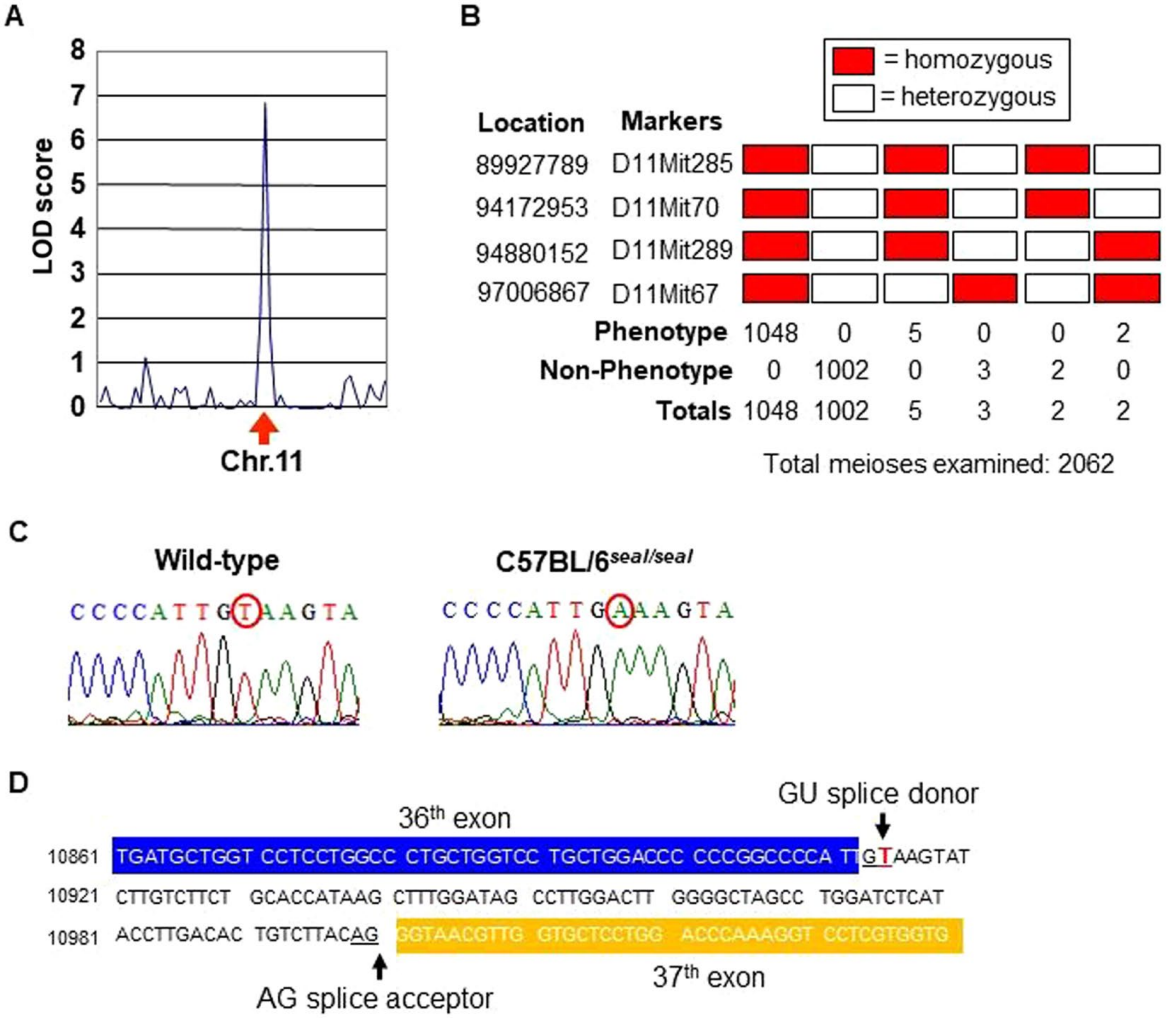
Genetic mapping and identification of the *seal* mutation. (**A**) The *seal* mutation was mapped to chromosome 11 on 34 meioses with peak LOD score of 6.9. Phenotypic classification was based on the inducible defect in hind limb movement. (**B**) Fine mapping of the mutation on chromosome 11 using microsatellite markers. The *seal* mutation was confined to a critical region marked by D11mit289 and D11mit67 with five crossover events proximal and three crossovers distal to the mutation. (**C**) DNA sequence chromatograms of the region containing the *seal* mutation. (**D**) Illustration of the position of the *seal* mutation relative to exons 36 and 37 of *Col1a1*.

The mutation causing the *seal* phenotype was mapped to chromosome 11 by genome-wide linkage analysis using a panel of 59 microsatellite markers (Fig. 2A); 4 additional chromosome 11 markers were used to confine the mutation to a 2.1 Mbp critical region containing 81 genes (Fig. 2B). Residing in the critical region, *Col1a1* encoding the type I collagen, α1 chain was considered a promising candidate since type I collagen mutations generally result in bone fragility^15^. Sequencing of *Col1a1* identified a single nucleotide transversion (T → A) in the donor splice site of intron 36 at position + 2 relative to the exon 36 boundary (Fig. 2C,D).

### Defective bone structure, turnover, and collagen network in *seal* mice

**Figure 3:**
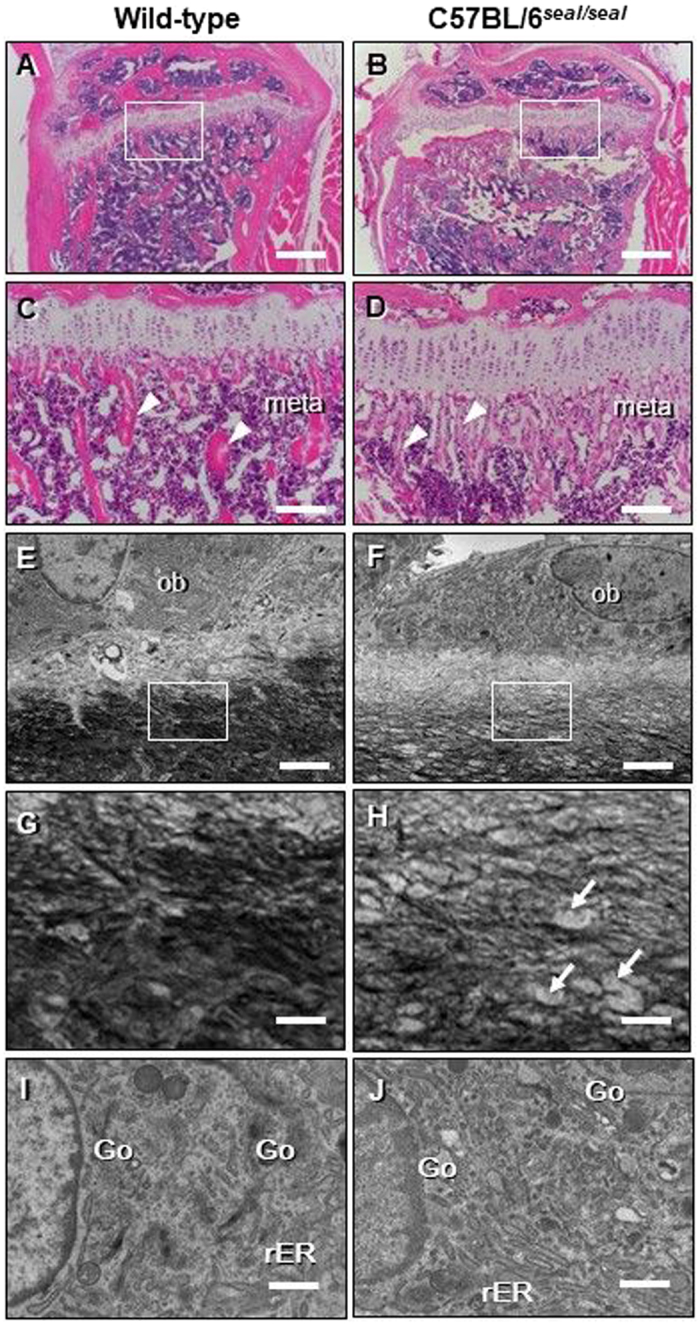
Abnormal bone structure and collagen network in *seal* homozygotes. Light microscopic (**A–D**) or TEM imaging (**E–J**) of the tibial sections from wild-type and *seal* homozygous mice. (**A**, **B**) The proximal region of the tibia. (**C**, **D**) Enlargement of the regions boxed in A and B, the metaphysis. Many thin trabeculae (arrowheads in **D**) were found in *seal* metaphysis (meta) compared with those (arrowheads in **C**) in wild-type mice. (**E, F**) Osteoblasts (ob) of the tibia. (**G**, **H**) Enlargement of the regions boxed in E and F, the bone matrix. Wild-type bone matrix demonstrated dense collagen fibrils (black fibrillar structures), whereas *seal* bone matrix contained sparsely-distributed collagen fibrils featuring organic materials (arrows). (**I**, **J**) Both wild-type and *seal* homozygous osteoblasts in the metaphyses of tibiae were cuboidal in shape, and showed developed rough endoplasmic reticulum (rER) and Golgi apparatus (Go). Scale bars, A, B: 500 μm, C, D: 100 μm, E, F: 2 μm, G-J: 0.5 μm.

**Figure 4:**
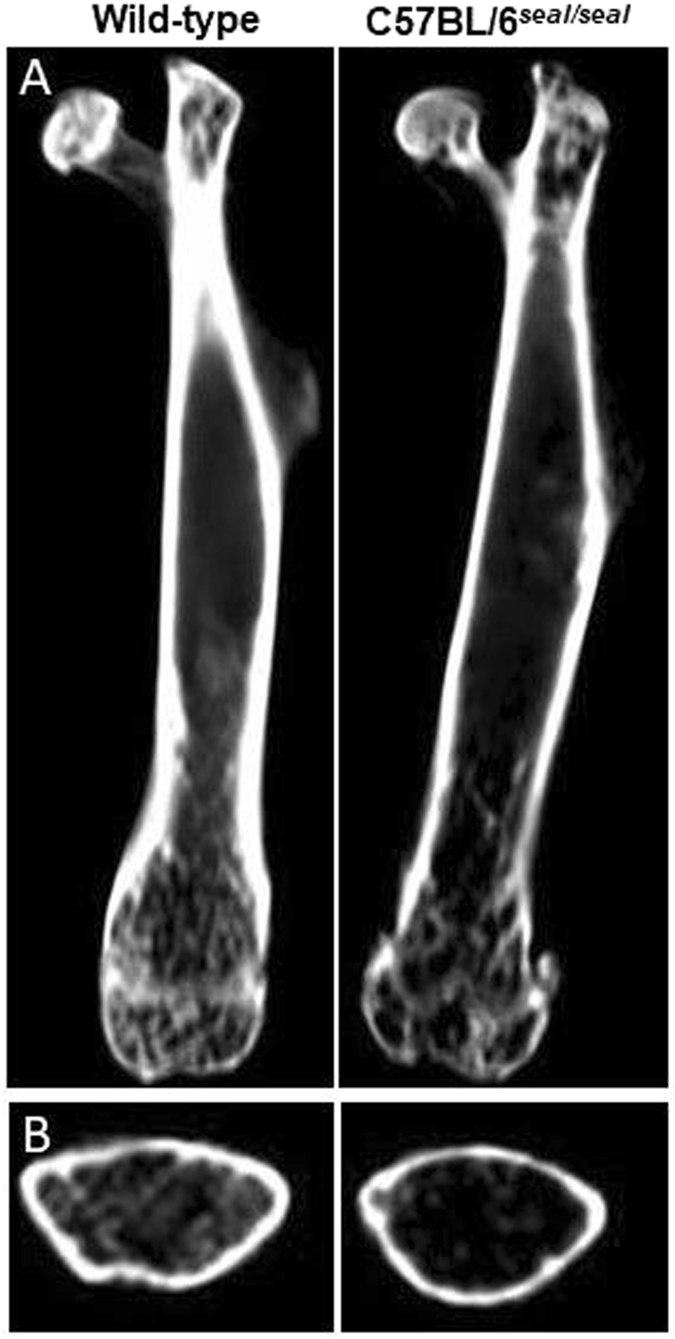
Thin cortical bone in *seal* femurs. Representative micro-CT images for wild-type mice (left) and *seal* homozygotes (right). (**A**) Sagittal sections. (**B**) Cross section of mid-diaphysis.

**Figure 5:**
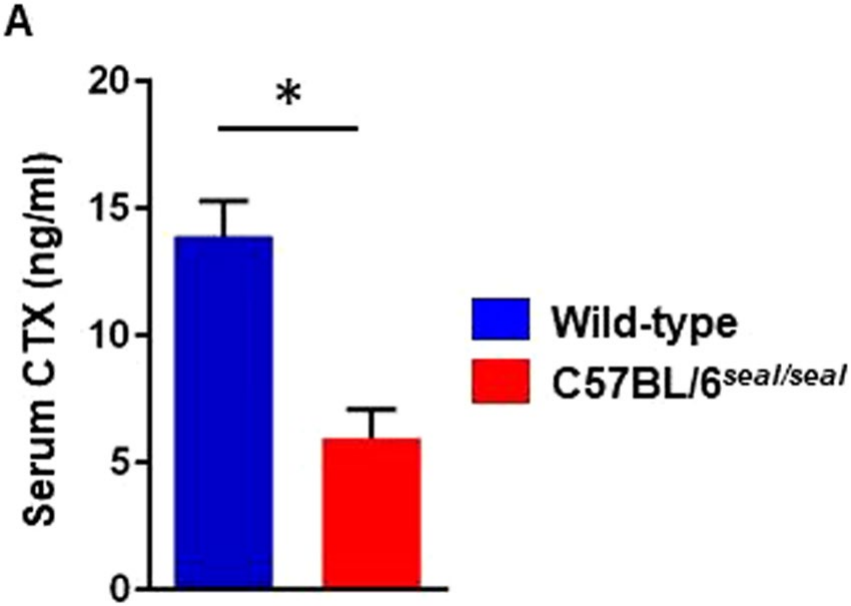
Metabolic balance of type I collagen in bone. CTX level in serum. N = 3 mice per genotype.

Type I collagen is a key structural component of bone, and we therefore examined bone structure in *seal* homozygotes. Hematoxylin and eosin staining revealed fine trabecular bone in the metaphysis of *seal* tibiae compared to that in wild-type controls (Fig. 3A–D), however, the trabecular number was not markedly different between the two groups by gross visual evaluation. Moreover, artificial fissures indicative of bone fragility were often discovered in the bone sections of *seal* homozygotes but not in those of wild-type mice (Fig. 3A,B). Micro-computed tomography (CT) images showed thinner cortical bone in *seal* femurs in comparison with wild-type femurs (Fig. 4). Assessment of trabecular and cortical parameters revealed significant defects in *seal* mice compared to wild-type controls. For example, cortical thickness and cortical bone area fraction were reduced 14% (*P* = 0.0016) and 24% (*P* < 0.0001), respectively, in *seal* homozygotes relative to wild-type mice. *Seal* mice also showed a significant decrease of trabecular bone mineral density (BMD; *P* = 0.0002), in agreement with the histological observation of fine trabeculae with reduced bone deposition (Table 1). Transmission electron microscopy (TEM) demonstrated many intact osteoblasts containing well-developed cellular organelles such as rough endoplasmic reticulum and Golgi apparatus, indicating the active form of osteoblasts in both wild-type and *seal* tibiae (Fig. 3E,F,I,J). The wild-type bone matrix showed densely-deposited collagen fibrils (See black fibrillar structures in Fig. 3G), while *seal* bone matrix contained sparsely-distributed collagen fibrils, consequently including many foci of organic materials (arrows in Fig. 3H). The effect of the *seal* mutation on steady state bone resorption *in vivo* was determined by measurement of the serum level of the type I collagen α1 chain C-terminal telopeptide (CTX), a biomarker for osteoclast activity. CTX levels in *seal* mice were reduced approximately 65% compared to those in wild-type mice (Fig. 5).

**Table 1:** Trabecular and cortical parameters from micro-CT analysis.

Parameters	Wild-type		*seal*		
	Mean	SD	Mean	SD	*P* value
Cortical BMD^a^ (mg/cm^3^)	983.225	18.442	975.175	9.879	0.4780
Trabecular BMD^a^ (mg/cm^3^)	161.85	26.65	16.825	4.325	0.0002^**^
Cortical thickness (mm)	0.196	0.007	0.168	0.003	0.0016^*^
Mean total cortical bone area^b^ (mm^2^)	1.051	0.052	1.037	0.031	0.7283
Mean total tissue area^b^ (mm^2^)	2.572	0.064	3.317	0.049	<0.0001^**^
Mean total tissue perimeter^b^ (mm)	6.359	0.089	7.161	0.066	<0.0001^**^
Cortical bone area fraction (%)	0.409	0.012	0.312	0.007	<0.0001^**^

^a^Bone mineral density. ^b^Crossectional area was analyzed. N = 4 mice per genotype.

**Figure 6:**
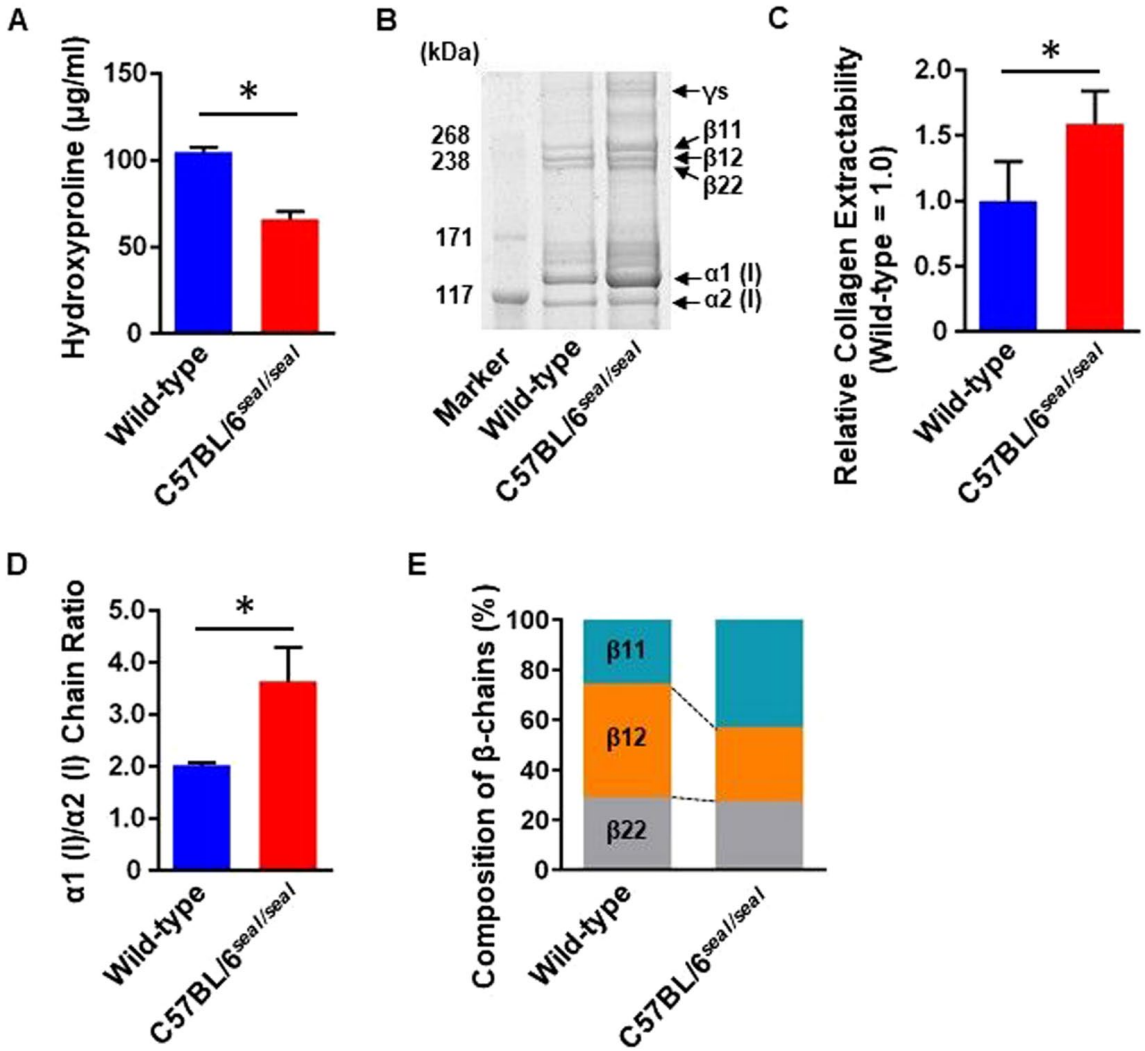
Collagen quantification and component assay. (**A**) Hydroxyproline content in demineralized bone hydrolysate was measured as an indicator of collagen content. (**B**) Representative gel image of femur type I collagen components separated by SDS-PAGE and visualized by CBB. For β-chains, numbers following the β designation indicate the identity of the two α-chain components [e.g. β12 is a heterodimer of α1 (I) and α2 (I)]. Band intensity represents collagen extractability. (**C**) Gel bands from B were quantitated by densitometric image analysis. Sum of quantitated band intensities of all type I collagen chains, representing collagen extractability, was plotted (normalized to wild-type). (**D**) Ratio of band intensities of α1 (I) and α2 (I) chains (α1 (I)/α2 (I) chain ratio). (**E**) Quantitation of β-chains by densitometric image analysis of CBB-stained SDS-PAGE gel containing type I collagen components from an independent extraction from femur samples. N = 3 mice per genotype. Data are expressed as mean ± SEM.

Type I collagen biosynthesis is a complex process that includes multiple post-translational modifications (e.g. hydroxylation of proline) leading to the formation of covalently cross-linked collagen fibrils^18^. To understand the effects of collagen mutations, it is important to analyze collagen quantity as well as collagen components, which are crucial determinants of bone mechanical properties^19^. To analyze the effect of the *seal* mutation on the collagen amount in bone, hydroxyproline content was evaluated in demineralized bone hydrolysate^20^. The bone samples from *seal* homozygotes contained significantly less hydroxyproline indicating reduced collagen content compared with wild-type bone (Fig. 6A). These data support the conclusion that the *seal* phenotype is due to reduced collagen, rather than accelerated bone turnover.

To examine the composition of type I collagen in bone from *seal* mice, collagen components from femurs were extracted, separated by SDS-PAGE, and visualized by CBB staining (Fig. 6B and Supplementary Fig. S1). Band intensities were higher for *seal* bone samples, reflecting increased collagen extractability relative to wild-type bone samples, which was supported by quantitation using densitometric image analysis (Fig. 6C). The quantitation also revealed an α1 (I)/α2 (I) chain ratio of 2.0 in control mice, reflecting the formation of a heterotrimer of two α1 (I) chains and one α2 (I) chain. Notably, the α1 (I)/α2 (I) chain ratio in *seal* mice was elevated to 3.6, suggesting the formation of some α1 (I) homotrimers (Fig. 6D).

β-chains refer to α-chain dimers in which the two α-chains are linked by intra- or intermolecular covalent cross-links, which remain intact under the conditions of SDS-PAGE while non-covalent bonds of the type I collagen triple helix are destroyed. We analyzed the composition of such dimers based on the distinct migration in SDS-PAGE of each possible dimer. In control mice, β12 chains [heterodimers of α1 (I) and α2 (I)] predominated, whereas β11 chains were the major form in *seal* mice, consistent with the possible formation of α1 (I) homotrimers in *seal* mice (Fig. 6E). These findings indicate that both the amount and the composition of collagen were altered in *seal* homozygous mice.

### Reduced *Col1a1* transcripts in *seal* mice but normal splicing

**Figure 7:**
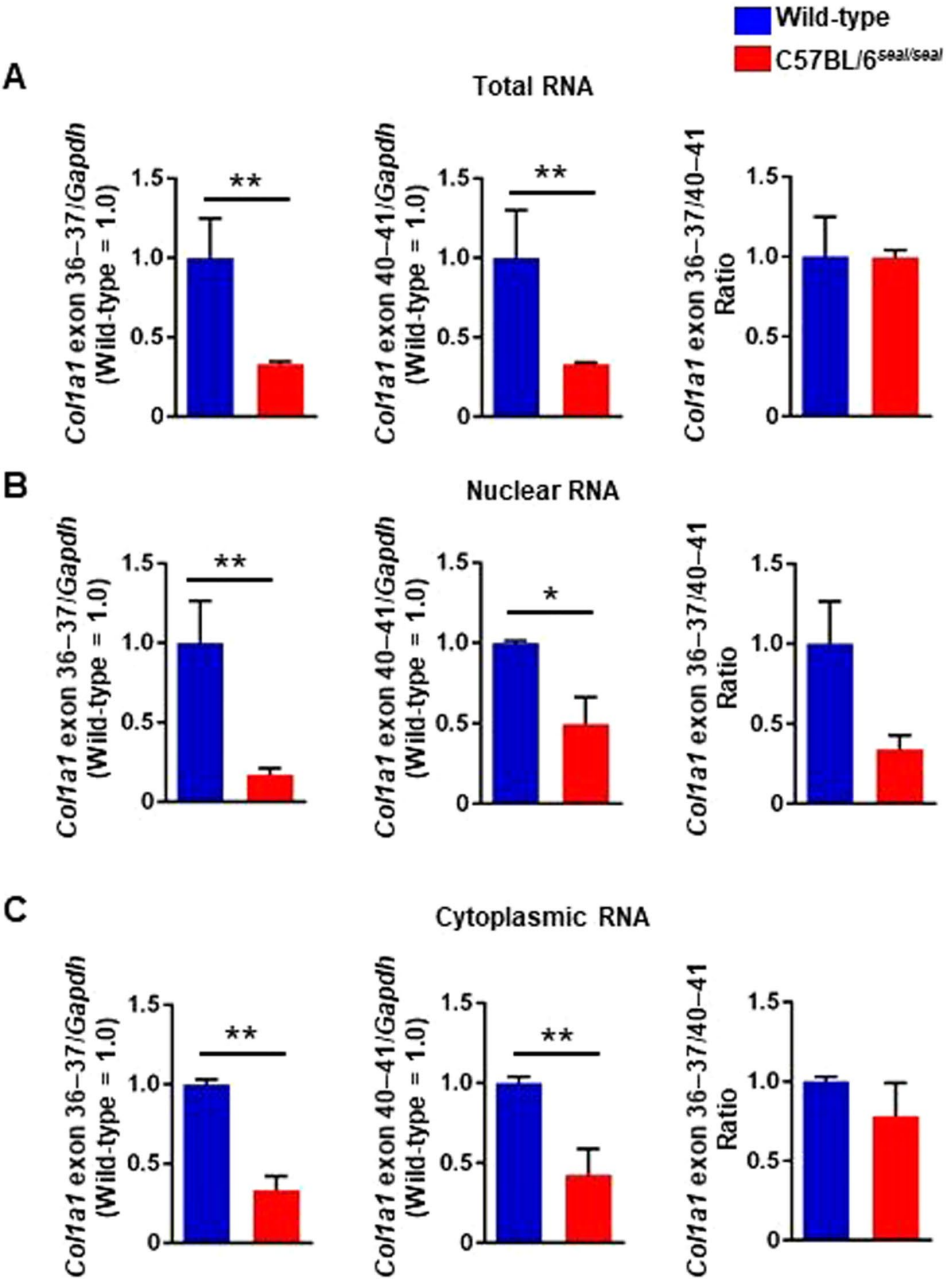
Reduced type I collagen gene expression in *seal* mice. *Col1a1* gene expression relative to *Gapdh* in the femur were measured by quantitative RT-PCR using primer sets targeting sequences in exons 36 and 37, or exons 40 and 41. The ratio of *Col1a1* exon 36–37/40–41 was analyzed for total RNA (**A**), nuclear RNA (**B**), and cytoplasmic RNA (**C**). *P*-values, (**A**) *P* = 0.007, *P* = 0.002, and n.s. from left, (**B**) *P* = 0.036, *P* = 0.040, and n.s. from left, and (**C**) *P* = 0.002, *P* = 0.027, and n.s. from left. N = 3 mice per genotype. Data are expressed as mean ± SEM.

Donor splice site mutations often result in exon skipping, and the *seal* mutation was predicted to cause skipping of the 108-nucleotide exon 36, resulting in an in-frame deletion of 36 amino acids near the middle of the α1 chain helical domain. Using quantitative RT-PCR, we confirmed that *Col1a1* transcripts were significantly reduced in total RNA, nuclear RNA, and cytoplasmic RNA from *seal* femurs relative to wild-type femurs based on two separate amplification analyses targeting exons 36–37 and 40–41 (Fig. 7A–C), consistent with the observation of reduced collagen fiber content in the bone matrix by TEM. However, the ratio of exon 36–37/exon 40–41 *Col1a1* mRNA in the nucleus, but not the cytoplasmic or total RNA, was reduced in *seal* mice relative to wild-type mice (Fig. 7A–C, right) due to greatly reduced levels of exon 36–37 transcript compared to exon 40–41 transcript in the nuclear fraction of *seal* femurs (Fig. 7B, left and middle).

**Figure 8:**
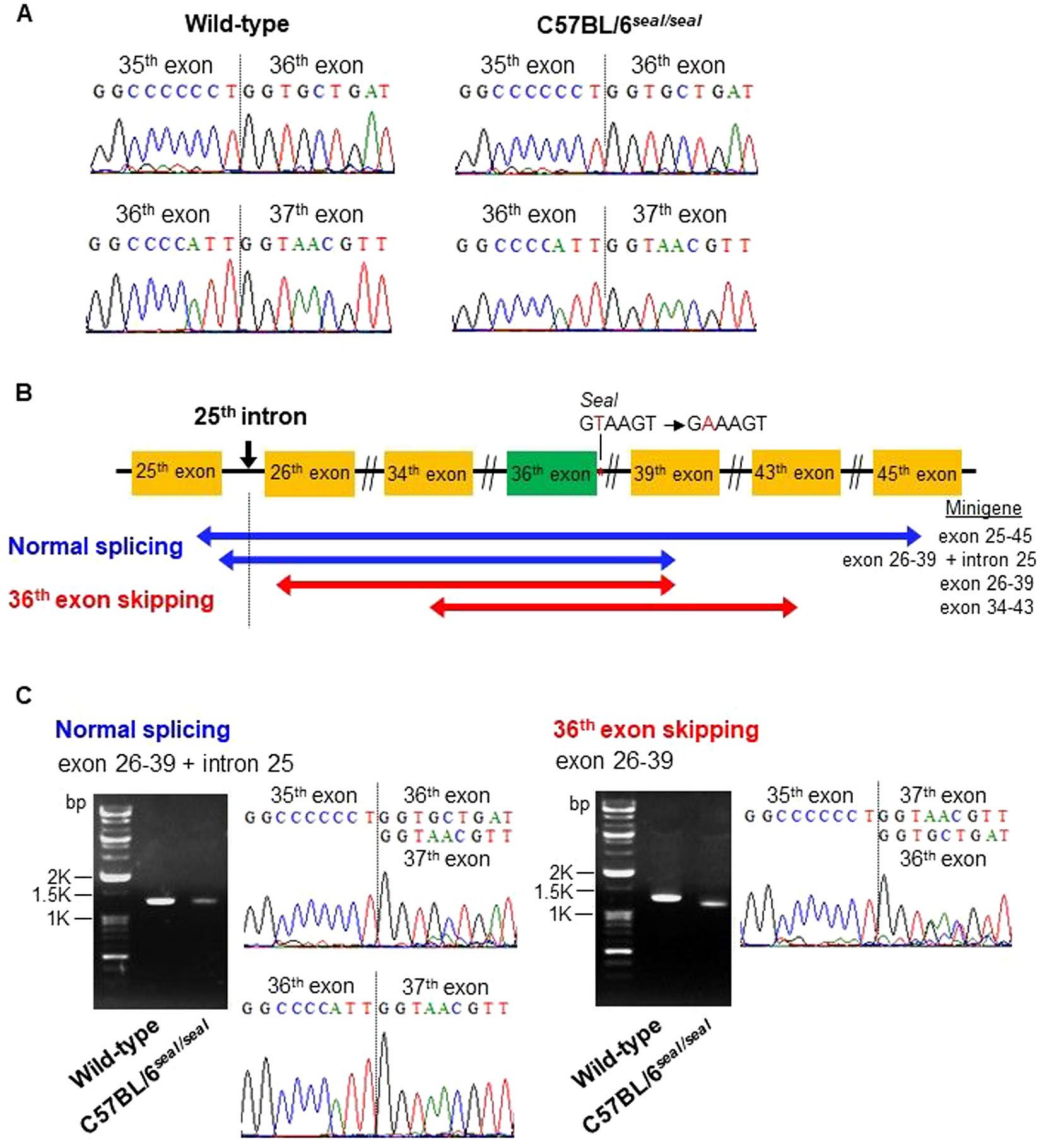
Minigene assay for *Col1a1* ^*seal*^ pre-mRNA splicing. (**A**) DNA sequence chromatograms of femur RT-PCR amplification products generated using primers complementary to sequences in exons 26 and 39. (**B**) Schematic illustration of *Col1a1* ^*seal*^ exons used in minigenes exon 25–45, 26–39 + intron 25, 26–39, and 34–43. Red and blue coloring denotes minigenes spliced without or with exon 36, respectively. (**C**) Sequence analyses and gel images of spliced transcripts of *Col1a1* ^*seal*^ minigene exon 26–39 (right). The major splice product from *Col1a1* ^*seal*^ minigene exon 26–39 lacked exon 36 and gel image showed corresponding band was shifted down compared with wild-type minigene band. In contrast, the majority of transcripts from *Col1a1* ^*seal*^ minigene exon 26–39 + intron 25 (left) were correctly spliced, with a minor fraction having exon 36 completely skipped which was not observed *in vivo*.

Sequencing of *Col1a1* transcripts across the junction between exon 35 and the next exon revealed normal splicing of exon 35 to exon 36 in RNAs isolated from *seal* femurs; normal splicing between exon 36 and exon 37 was also observed (Fig. 8A), although we cannot exclude the possibility that minor amounts of abnormal transcripts were present below the level of detection. The recessive nature of the *seal* phenotype suggests that the quantity of aberrant type I collagen α1 chains produced, if any, is insufficient to exert a dominant negative effect. These data suggest that the *seal* mutation slows the rate of splicing such that *Col1a1* mRNA levels are diminished in *seal* bone compared to those in wild-type bone, despite correct splicing of exon 35 to 36 and exon 36 to 37.

To examine the effect of the *seal* mutation on *Col1a1* pre-mRNA splicing, we constructed four *Col1a1* minigenes containing the *seal* mutation and assessed mRNA splicing following transfection into HEK293 cells (Fig. 8B). Minigenes exon 26–39 (Fig. 8C, right) and exon 34–43 (Fig. S2, right) yielded transcripts in which exon 36 was mostly skipped; we noted that normally spliced transcripts of these minigenes were detected by DNA sequencing but not by staining in gels, likely due to a low abundance of normally spliced transcripts below the level of detection by ethidium bromide staining. In contrast, minigene exon 25–45 (Fig. S2, left) produced properly spliced transcripts. We hypothesized that the presence of intron 25 promoted inclusion of exon 36 in the spliced minigene mRNAs. To test our hypothesis, we constructed a minigene in which intron 25 was included in minigene exon 26–39 (exon 26–39 + intron 25; Fig. 8B), and examined splicing upon expression in HEK293 cells. Addition of intron 25 promoted inclusion of exon 36 in the majority of transcripts, although residual transcripts lacking exon 36 were still produced (Fig. 8C, left). These data suggest that *Col1a1* intron 25 may contain elements that compensate for the splicing error caused by the splice site mutation and support normal splicing of exon 36.

## Discussion

The helical domain of murine type I collagen α1 chain is encoded by 43 of the 51 total exons of *Col1a1*; these 43 exons encode the repeating sequence Gly-X-Y, and each begins with a glycine codon and ends with a Y-position codon. Because a glycine residue at every third position of the chain is critical to the formation of the triple helix of mature type I collagen^21^, frameshifts or premature termination caused by aberrant splicing can be detrimental for type I collagen synthesis^15,16^. We speculate that multiple redundant mechanisms have evolved to ensure proper splicing of collagen mRNAs. In support of this hypothesis, only normal *Col1a1* transcripts were detected in bone tissue of *seal* mice, despite the presence of a mutation in the invariant GU dinucleotide of the intron 36 donor splice site. Results of splicing analyses using minigenes suggested that either an intact 5′ss in intron 36 or the presence of a putative intronic splicing enhancer in intron 25 was necessary for proper splicing of exon 36 in minigene 26–39. An implication of this finding is that removal of intron 36 precedes removal of intron 25. However, the intron 25 regulatory element appeared to be less efficient than the 5′ splice site of intron 36 in directing splicing, as evidenced by the overall reduction in *Col1a1* transcript abundance in bone from *seal* mice relative to wild-type mice, which was also observed in the minigene analysis. The observation of a low frequency of spliced transcripts lacking exon 36 among intron 25-containing *seal* minigene transcripts (Fig. 8C, left), as well as a low frequency of normally spliced *seal* minigene transcripts lacking intron 25 (Fig. 8C, right), suggests that other cis-acting splicing regulatory elements outside intron 25, or trans-acting regulators, also contribute to normal exon 36 splicing. Further studies are necessary to identify the critical element(s) in *Col1a1* intron 25 and their mechanism(s) of action in supporting proper splicing of exon 36 and possibly other exons.

A comparison of the nuclear and cytoplasmic abundance of *Col1a1* transcripts as measured across the exon 36–37 junction versus the exon 40–41 junction revealed a reduction of the ratio of exon 36–37/exon 40–41 *Col1a1* mRNA in the nuclear fraction of *seal* femurs relative to wild-type femurs, whereas similar exon 36–37/exon 40–41 ratios were found in *seal* and wild-type cytoplasmic fractions. These data suggest that the effect of the *seal* mutation may be to slow down splicing of the exon 36–37 junction compared to the exon 40–41 junction, resulting in greatly reduced levels of *Col1a1* exon 36–37 transcript compared to exon 40–41 transcript in nuclei of *seal* femurs. In the cytoplasm, the difference was reduced, presumably because only fully spliced transcripts are allowed to exit the nucleus; hence the ratio of exon 36–37/exon 40–41 *Col1a1* mRNA in the cytoplasm of *seal* femurs is not significantly different from the ratio in wild-type femurs.

Human OI manifests a wide spectrum of severity as well as variability of causative mutations. Administration of bisphosphonates was shown to effectively increase vertebral areal bone mineral density and height. However, concerns have been raised as to its efficacy for fracture reduction^22–25^. This suggests that understanding the relationship between clinical manifestation and underlying pathogenesis is necessary for the development of effective therapy, and animal models mimicking various types of human OI are pivotal to these studies. *Seal* mutant mice, showing short limbs with short undermineralized long bones and sporadic limb deformity, model human type III OI. *Oim*, a spontaneous mutation of *Col1a2* encoding the type I collagen pro-α2 chain^26^, causes a phenotype similar to *seal*. However, the underlying mechanisms are different. The *oim* mutation causes aberrant pro-α2 (I) collagen synthesis that inhibits assembly of a normal type I collagen trimer^26,27^. In contrast, type I collagen in *seal* mice was greatly reduced due to a decrease in transcription of the α1 chain, and that which was produced consisted, at least in part, of α1 (I) homotrimers, which have been associated with impaired bone strength leading to increased risk of bone fracture^26,28–31^. In normal type I collagen, the hydrophobicity of the α2 (I) chain is thought to promote the stability of the heterotrimer by increasing the hydrophobic interactions between the heterotrimeric molecules, and increasing the binding of the molecules in the fiber^32^. Therefore, the elevated α1 (I)/α2 (I) chain ratio of type I collagen from *seal* mice may signify a reduced efficiency of self-assembly and loose-packing collagen fibers. In addition, it has been shown that each tissue has a unique collagen cross-link pattern that supports the tissue′s mechanical features. An abnormal pattern of collagen cross-linking is often observed in aged and diseased bone, making it brittle or fragile^33^. Our results showed that the composition of β-chains differed between type I collagen in bone from *seal* versus wild-type mice; this abnormal collagen cross-link pattern may also contribute to decreased fracture strength of bone in *seal* mice. *Seal* mice provide a valuable disease model of human OI, in which *Col1a1* splicing regulation and its effects on transcript and protein abundance, and on type I collagen fiber formation may be investigated.

## Methods

### Mice

C57BL/6 J and C3H/HeN mice were obtained from The Jackson Laboratory (Bar Harbor, ME) and Taconic Biosciences (Germantown, NY) and maintained under specific pathogen-free conditions in The Scripps Research Institute vivarium and Niigata University animal facility. All male mice used in the experiments were 4–12 weeks in age. Animals were to be excluded from analysis only if they displayed obvious illness or death; these conditions were not observed and no animals were excluded. No randomization of the allocation of animals to experimental groups was performed.

### Data availability

All data generated or analyzed during this study are included in this published article (and its Supplementary Information files). The *seal* strain (*Col1a1* ^*m1Btlr*^; MGI: 3776559) is described at http://mutagenetix.utsouthwestern.edu and is available from the Mutant Mouse Regional Resource Center (MMRRC: 030348-UCD).

### Ethics Statement

All experimental procedures using mice were approved by and conducted in accordance with The Scripps Research Institute Institutional Animal Care and Use Committee, and Niigata University institutional guidelines for animal care and use. The protocol to perform euthanasia by cervical dislocation after intraperitoneal injection of chloral hydrate and to obtain specimens was approved by the animal ethics committee for animal experimentation of Niigata University (Permit Number: 39). Any unnecessary grasping of *seal* homozygous mice by the scruff of the neck was avoided and all efforts were made to minimize suffering.

### ENU Mutagenesis, phenotypic screens, and linkage analysis

Random germline mutagenesis of C57BL/6 J mice using ENU was described previously^34^. Phenotypic screening including was applied to G3 and G1 mice. Phenotypic screens included casual inspection for immunodeficiency and dysmorphologies affecting limbs, tail, eyes, teeth, or other aspects of body form; coat color and/or coat quality anomalies, abnormal body size^35–40^. Homozygous *seal* mice were mated to wild-type C3H/HeN mice, and their progeny were backcrossed to the homozygous mutant stock. 34 F2 mice were scored for phenotype and genomic DNA was prepared from tail tips for genotyping. 59 microsatellite markers were used for genome-wide linkage analysis.

### *In vitro* pre-mRNA splicing assay

*Col1a1* mRNA processing was analyzed using a minigene assay. Briefly, *Col1a1* exon 25–45, 26–39 + intron 25, 26–39, and 34–43 were amplified from genomic DNAs prepared from wild-type and homozygous *seal* mice and cloned into vector pcDNA3.1/V5-His-TOPO (Invitrogen, Carlsbad, CA) as minigene constructs. Primers are listed in Supplementary Table S1. HEK293 cells (DS Pharma Biomedical Co., Ltd, Osaka, Japan) transiently transfected with purified minigene plasmids were harvested 48 h post transfection, and then total RNA was extracted using TRIzol® reagent (Invitrogen). The RNA was reverse transcribed by M-MLV reverse transcriptase (Invitrogen) in to cDNA using random primers (Takara Bio Inc., Shiga, Japan). Sequence analysis was performed using primer covering exon 36 and 37 to analyze the exon skipping.

### Quantitative reverse transcription RT-PCR

Femurs RNA was extracted using TRIzol® reagent (Invitrogen) to obtain total RNA or cytoplasmic and nuclear RNA purification kit (Norgen, ON, Canada) to obtain cytoplasmic and nuclear RNA according to the manufacturer’s instructions. Total RNA (1 μg) was reverse transcribed by M-MLV reverse transcriptase (Invitrogen) using random primers (Takara Bio Inc., Shiga, Japan). Grinded specimens for femurs RNA extraction were prepared using SK-mill, after removing bone marrow and subsequent deep freezing in liquid nitrogen. TaqMan Probe® Mm01302046_g1 (Exon 36–37) and Mm00801658_g1 (Exon 40–41) for real-time PCR were purchased from Applied Biosystems (Foster City, CA). Reactions were carried out in the ABI PRISM 7900HT Sequence Detection System (Applied Biosystems) using TaqMan Gene Expression Assays (Applied Biosystems) containing 900 nM primer and 250 nM probe in a 25 µl mixture. The reactions consisted of a 10 min incubation at 95 °C, followed by 40 cycles of a two-step amplification procedure of annealing/extension at 60 °C for 1 min and denaturation for 15 s at 95 °C. ABI PRISM SDS 2.0 software (Applied Biosystems) was used to carry out the quantifications. The relative quantity of each mRNA was normalized to glyceraldehyde-3-phosphate dehydrogenase (*Gapdh*) mRNA.

### Histological Analysis

12-week-old *seal* mice and their wild-type littermates were fixed with 4% paraformaldehyde in a 0.1 M phosphate buffer (pH 7.4) under appropriate anesthesia (intraperitoneal injection of chloral hydrate). Tibiae were removed *en bloc* and immediately immersed in the same fixative for 12 h, then decalcified with 10% ethylenediamine tetraacetic disodium salt (EDTA-2Na) solution for light microscopic observation for 4 weeks, or 5% EDTA-2Na solution for electron microscopic analysis for 6 weeks. Staining of tissue sections were carried out using hematoxylin-eosin for light microscopy. Some undecalcified tibiae were post-fixed with 1% osmium tetroxide with a 0.1 M cacodylate buffer for 4 h at 4 °C, dehydrated in ascending acetone solutions, and embedded in epoxy resin (Epon 812, TAAB Laboratories Equipment Ltd., Berkshire, UK). Ultrathin sections prepared with an ultramicrotome, were stained with 1% tannic acid, uranyl acetate and lead citrate for observation under transmission electron microscope (Hitachi H-7100; Hitachi Co. Ltd, Tokyo, Japan) at 80 kV.

### Micro-CT analysis

Micro-computed tomographic scans were performed on excised femurs and morphological analysis was performed using micro-CT (SkyScan 1174, Bruker microCT, Kontich, Belgium) with an X-ray tube voltage of 50 kV and current of 800 μA, as described by Cano *et al*.^41^. The angular rotation was 185°, and the angular increment was 0.45° for scanning. The voxel size was set at 6.5 μm isotropically. A modified Feldkamp algorithm was used for reconstruction of data sets and segmentation into binary images (8-bit BMP images) was carried out using adaptive local thresholding. The microarchitectural properties of trabecular and cortical bone regions were evaluated within a conforming volume of interest (VOI). A VOI in the trabecular bone region was started at a distance of 1 mm from the distal growth plate, extending a further 2 mm of longitudinal distance in the proximal direction (96 image slices). The regions of trabecular bone were consisted of cylindrical segments (radius 0.86 mm). A VOI included in the middiaphyses (96 images) was selected in the cortical bone region. Cortical bone regions were selected by free drawing regions of interest. Cortical thickness (mm), mean total crossectional cortical bone area (mm^2^), mean total crossectional tissue area (mm^2^), mean total crossectional tissue perimeter (mm), cortical bone area fraction (%) were analyzed. Cortical bone mineral density (Cortical BMD) and trabecular bone mineral density (Trabecular BMD) were calculated using the conforming VOI. Reconstructed 8-bit BMP images have a grey value between 0 and 255 in every pixel. 255 was assumed to be white (void space), whereas 0 is black, the densest part of the image. Hydroxyapatite phantom rods (2 mm of diameter) immersed in pure water, equivalent to BMD of 0.25 g/cm^3^ and 0.75 g/cm^3^ were employed for calibration to express grey values as mineral content.

### Metabolism of bone type I collagen

Degradation of bone type I collagen was evaluated, measuring C-terminal cross-linking type I collagen fragments (CTX) in serum by RatLaps™ (CTX-I) ELISA kit (Immunodiagnostic Systems Limited, Boldon, UK) according to the manufacturer’s protocol.

### Quantification and qualification of collagen

Quantification of collagen contents in femurs was evaluated by hydroxyproline assay. Femurs were collected from 10-week-old mice. After completely remove the connective tissues, both ends of femurs were cut off, and bone marrow was washed out by ice cold phosphate buffer saline. The cleaned bone samples were demineralized with 10% EDTA for 1 week, dialyzed against water using Spectra/Por (MWC 3,500 Da, Spectrum Laboratories, Inc., Milipitas, CA) for 4 days, and lyophilized. Sample preparation was performed below 4 °C unless otherwise specified. Equal weight of samples were hydrolyzed by 12 M HCl for 20 h at 95 °C. Quantification of hydroxyproline, representing total collagen amount, was performed by a Total collagen assay kit (QuickZyme Biosciences, Leiden, Netherlands), according to the manufacturer’s instruction.

Collagen components were analyzed using demineralized and lyophilized bone samples. Samples of equal weight were directly resolved in sodium dodecyl sulfate (SDS) sample buffer (Life Technologies, Carlsbad, CA), heated for 10 min at 80 °C, and centrifuged at 13,000 × g for 20 min. Equal volume of supernatants were loaded onto the NuPAGE 3–8% Tris-Acetate Gel (Life Technologies), and the electrophoresis was performed at constant voltage of 150 V for 60 min. Gels were stained with Coomassie Brilliant Blue R (CBB, Sigma-Aldrich, St Louis, MO). Digital images were taken by Image Scanner GT-X970 (Epson, Nagano, Japan). Each band, corresponding α-, β- and γ-chains of type I collagen were quantified by ImageJ software. Collagen extractability (α + β + γ), α1/α2 chain ratio and composition ratio of β-chains were calculated.

### Statistical analysis

Comparisons of differences were between two unpaired experimental groups in all cases. An unpaired *t*-test (Student’s *t*-test) is appropriate and was used for such comparisons. The phenotypic performance of mice (C57BL/6J) is expected to follow a normal distribution, as has been observed in large datasets from numerous phenotypic screens conducted by our group. Variation within each dataset obtained by measurements from mice was assumed to be similar between genotypes since all strains were generated and maintained on the same pure inbred background (C57BL/6J); experimental assessment of variance was not performed.

The statistical significance of differences between experimental groups was determined using GraphPad Prism 5 (GraphPad Software Inc., La Jolla, CA,) and the Student’s *t*-test (unpaired, two-tailed). *P* < 0.05 was considered statistically significant and indicated by *P < 0.05 and **P < 0.001. No pre-specified effect size was assumed, and in general 3–5 animals or replicates for each genotype or condition were used in experiments; this sample size was sufficient to demonstrate statistically significant differences in comparisons between two unpaired experimental groups by unpaired *t*-test. The investigator was not blinded to genotypes or group allocations during any experiment.

## Electronic supplementary material

Supplementary Information

## References

[1] Nold JG, Kang AH, Gross J (1970). Collagen molecules: distribution of alpha chains. Science.

[2] Marini JC (2007). Consortium for osteogenesis imperfecta mutations in the helical domain of type I collagen: regions rich in lethal mutations align with collagen binding sites for integrins and proteoglycans. Hum Mutat.

[3] Van Dijk FS, Sillence DO (2014). Osteogenesis imperfecta: clinical diagnosis, nomenclature and severity assessment. Am J Med Genet A.

[4] Forlino A, Cabral WA, Barnes AM, Marini JC (2011). New perspectives on osteogenesis imperfecta. Nat Rev Endocrinol.

[5] Bodian DL (2009). Mutation and polymorphism spectrum in osteogenesis imperfecta type II: implications for genotype-phenotype relationships. Human molecular genetics.

[6] Peng H (2012). A novel splicing mutation in COL1A1 gene caused type I osteogenesis imperfecta in a Chinese family. Gene.

[7] D’Alessio M, Bernard M, Pretorius PJ, de Wet W, Ramirez F (1988). Complete nucleotide sequence of the region encompassing the first twenty-five exons of the human pro alpha 1(I) collagen gene (COL1A1). Gene.

[8] Bateman JF, Chan D, Moeller I, Hannagan M, Cole WG (1994). A 5’ splice site mutation affecting the pre-mRNA splicing of two upstream exons in the collagen COL1A1 gene. Exon 8 skipping and altered definition of exon 7 generates truncated pro alpha 1(I) chains with a non-collagenous insertion destabilizing the triple helix. Biochemical Journal.

[9] Wahl MC, Will CL, Luhrmann R (2009). The spliceosome: design principles of a dynamic RNP machine. Cell.

[10] Sheth N (2006). Comprehensive splice-site analysis using comparative genomics. Nucleic Acids Research.

[11] Roca X, Krainer AR, Eperon IC (2013). Pick one, but be quick: 5′ splice sites and the problems of too many choices. Genes and Development.

[12] Scotti MM, Swanson MS (2016). RNA mis-splicing in disease. Nat Rev Genet.

[13] Schwarze U, Starman BJ, Byers PH (1999). Redefinition of exon 7 in the COL1A1 gene of type I collagen by an intron 8 splice-donor-site mutation in a form of osteogenesis imperfecta: influence of intron splice order on outcome of splice-site mutation. American Journal of Human Genetics.

[14] Takahara K (2002). Order of intron removal influences multiple splice outcomes, including a two-exon skip, in a COL5A1 acceptor-site mutation that results in abnormal pro-alpha1(V) N-propeptides and Ehlers-Danlos syndrome type I. American Journal of Human Genetics.

[15] Kuivaniemi H, Tromp G, Prockop DJ (1991). Mutations in collagen genes: causes of rare and some common diseases in humans. FASEB Journal.

[16] Byers PH (1990). Brittle bones–fragile molecules: disorders of collagen gene structure and expression. Trends in Genetics.

[17] Forlino A, Marini JC (2016). Osteogenesis imperfecta. Lancet.

[18] Yamauchi M, Sricholpech M (2012). Lysine post-translational modifications of collagen. Essays in Biochemistry.

[19] Kuroshima, S. *et al*. A Paradigm Shift for Bone Quality in Dentistry: A Literature Review. *J Prosthodont Res*., in press (2017). 10.1016/j.jpor.2017.05.006 28633987

[20] Prockop DJ, Udenfriend S (1960). A specific method for the analysis of hydroxyproline in tissues and urine. Anal Biochem.

[21] Brodsky B, Persikov AV (2005). Molecular structure of the collagen triple helix. Adv Protein Chem.

[22] Rauch F, Glorieux FH (2004). Osteogenesis imperfecta. Lancet.

[23] Rauch F, Glorieux FH (2005). Osteogenesis imperfecta, current and future medical treatment. *American Journal of Medical Genetics*. Part C: Seminars in Medical Genetics.

[24] Castillo H, Samson-Fang L (2009). American Academy for Cerebral, P. & Developmental Medicine Treatment Outcomes Committee Review, P. Effects of bisphosphonates in children with osteogenesis imperfecta: an AACPDM systematic review. Developmental Medicine and Child Neurology.

[25] Ward LM (2011). Alendronate for the treatment of pediatric osteogenesis imperfecta: a randomized placebo-controlled study. Journal of Clinical Endocrinology and Metabolism.

[26] Chipman SD (1993). Defective pro alpha 2(I) collagen synthesis in a recessive mutation in mice: a model of human osteogenesis imperfecta. Proc Natl Acad Sci USA.

[27] Saban J (1996). Heterozygous oim mice exhibit a mild form of osteogenesis imperfecta. Bone.

[28] Mann V (2001). A COL1A1 Sp1 binding site polymorphism predisposes to osteoporotic fracture by affecting bone density and quality. J Clin Invest.

[29] Grant SF (1996). Reduced bone density and osteoporosis associated with a polymorphic Sp1 binding site in the collagen type I alpha 1 gene. Nat Genet.

[30] McBride DJ, Shapiro JR, Dunn MG (1998). Bone geometry and strength measurements in aging mice with the oim mutation. Calcif Tissue Int.

[31] Deak SB, van der Rest M, Prockop DJ (1985). Altered helical structure of a homotrimer of alpha 1(I)chains synthesized by fibroblasts from a variant of osteogenesis imperfecta. Collagen and Related Research.

[32] Miles CA, Sims TJ, Camacho NP, Bailey AJ (2002). The role of the alpha2 chain in the stabilization of the collagen type I heterotrimer: a study of the type I homotrimer in oim mouse tissues. J Mol Biol.

[33] Saito M, Marumo K (2015). Effects of Collagen Crosslinking on Bone Material Properties in Health and Disease. Calcified Tissue International.

[34] Hoebe K, Du X, Goode J, Mann N, Beutler B (2003). Lps2: a new locus required for responses to lipopolysaccharide, revealed by germline mutagenesis and phenotypic screening. Journal of Endotoxin Research.

[35] Arnold CN (2012). ENU-induced phenovariance in mice: inferences from 587 mutations. BMC research notes.

[36] Tabeta K (2004). Toll-like receptors 9 and 3 as essential components of innate immune defense against mouse cytomegalovirus infection. Proc Natl Acad Sci USA.

[37] Du X (2004). Velvet, a dominant Egfr mutation that causes wavy hair and defective eyelid development in mice. Genetics.

[38] Beutler B, Hoebe K, Georgel P, Tabeta K, Du X (2004). Genetic analysis of innate immunity: TIR adapter proteins in innate and adaptive immune responses. Microbes Infect.

[39] Tabeta K (2006). The Unc93b1 mutation 3d disrupts exogenous antigen presentation and signaling via Toll-like receptors 3, 7 and 9. Nat Immunol.

[40] Meehan TP (2006). Point mutations in the melanocortin-4 receptor cause variable obesity in mice. Mamm Genome.

[41] Cano A (2008). Comparative effects of 17beta-estradiol, raloxifene and genistein on bone 3D microarchitecture and volumetric bone mineral density in the ovariectomized mice. Osteoporosis International.

