# Supplementary material for: An ENU-induced splice site mutation of mouse Col1a1 causing recessive osteogenesis imperfecta and revealing a novel splicing rescue
Source: Sci Rep. 2017 Sep 15;7:11717. doi: 10.1038/s41598-017-10343-9 (PMC5600972; doi:10.1038/s41598-017-10343-9)
Supplement: Supplementary file 1 — Supplementary Information [file 41598_2017_10343_MOESM1_ESM.pdf]

An ENU-induced splice site mutation of mouse Col1a1 causing recessive osteogenesis imperfecta and revealing a novel splicing rescue

Koichi Tabeta, Xin Du, Kei Arimatsu, Mai Yokoji, Naoki Takahashi, Norio Amizuka, Tomoka Hasegawa, Karine Crozat, Tomoki Maekawa, Sayuri Miyauchi, Yumi Matsuda, Takako Ida, Masaru Kaku, Kasper Hoebe, Kinji Ohno, Hiromasa Yoshie, Kazuhisa Yamazaki, Eva Marie Y. Moresco, Bruce Beutler

Supplementary Fig. S1.

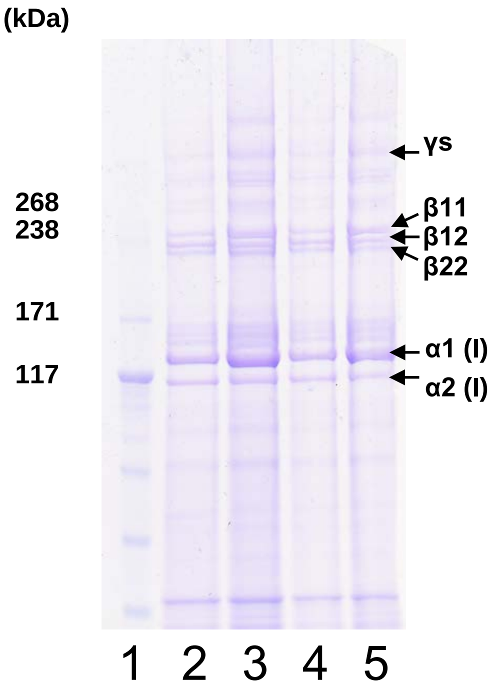

1.	Marker	
2.	Wild-type	5 µl
3.	C57BL/6$^{seal/seal}$	5 µl
4.	Wild-type	4 µl
5.	C57BL/6$^{seal/seal}$	4 µl

The full-length gel image for Figure 6B.
0.1 mg of demineralized freeze dried bone from wild-type and *seal* mice were lysed in 100 µl of SDS sample buffer. The indicated volumes were loaded on the gel.

Supplementary Fig. S2.

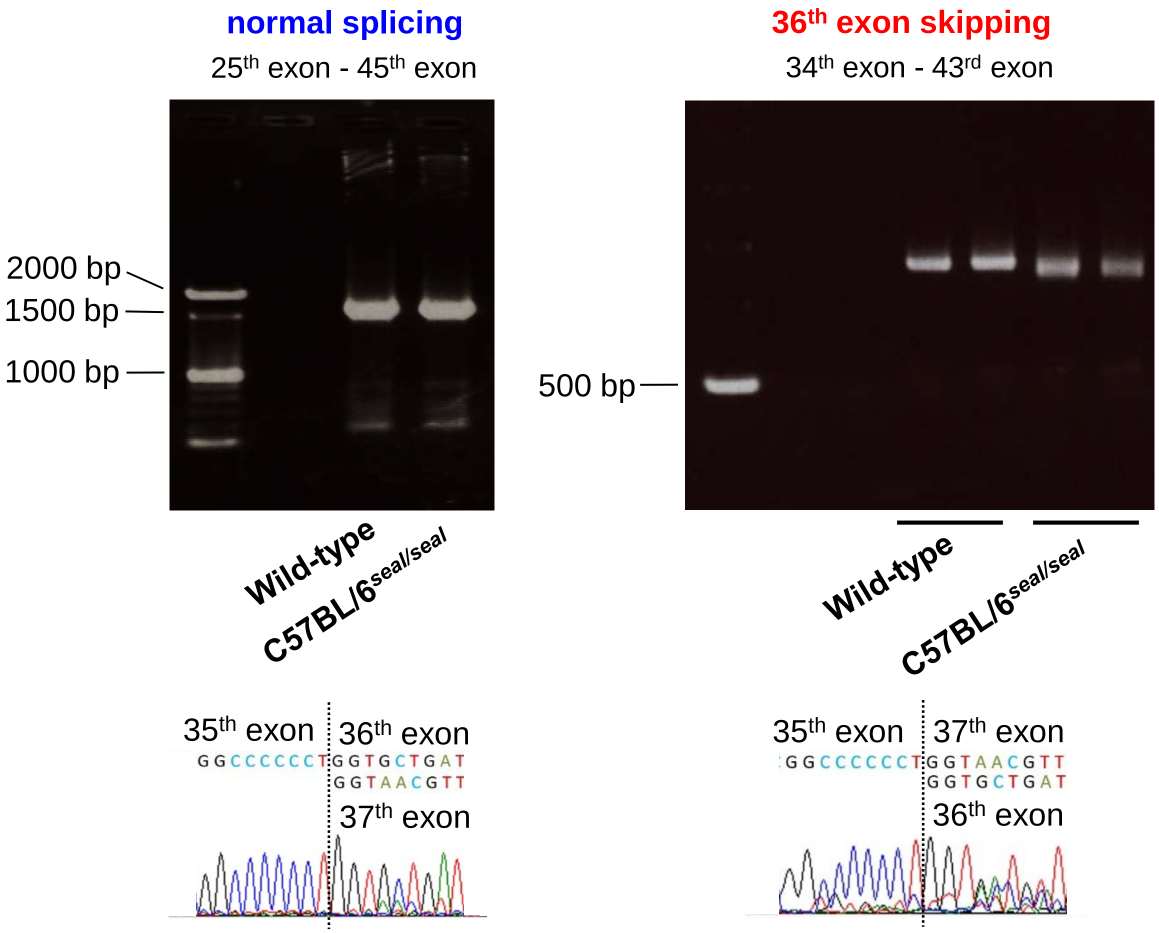

Sequence analyses and gel images of spliced transcripts of *Col1a1^{seal}* minigene exon 25-45 (left) and 34-43 (right). *Col1a1^{seal}* minigene exon 25-45 spliced correctly (left). The major splice product from minigene exon 34-43 lacked exon 36 and gel images of *Col1a1^{seal}* is shifted compared with wild-type.

Table S1. Primers for constructing minigenes

	Location	Sequence (Forward) 5'-3'	Location	Sequence (Reverse) 5'-3'
**exon 25-45**	7533-7553	atgggattccctggacctaag	13149-13127	actttatgcctctgtcaccttgtt
**exon 34-43**	10307-10326	atgaagctggtcccagtggtc	12538-12520	aaccacgatcgccattcttg
**exon 26-39 +intron 25**	7563-7590	agtgagtattcagtgaggaacactacaga	11407-11388	aggacctttgcccccttcttt
**exon 26-39**	8090-8109	aaacctggaaaggctggagag	11407-11388	aggacctttgcccccttcttt

Referenced sequence IDs for *Col1a1*: NM_007742.3 (transcript_id); MGI:88467 (gene_id)
